# A full-width half-maximum method to assess retinal vascular structural changes in patients with ischemic heart disease and microvascular angina

**DOI:** 10.1038/s41598-019-47194-5

**Published:** 2019-07-29

**Authors:** Bo Lun Xu, Wen Li Zhou, Tie Pei Zhu, Ke Yun Cheng, Yi Jie Li, Hai Jing Zhan, Li Gang Jiang, Yu Hua Tong

**Affiliations:** 1 Quzhou Central Hospital of Zhejiang Chinese Medical University, Quzhou People’s Hospital, Quzhou, Zhejiang Province China; 2 Department of Diagnostic Radiology, The University of Hong Kong, Hong Kong, SAR China; 3 Eye Center of Affiliated Second Hospital, Medical College of Zhejiang University, Hangzhou, Zhejiang Province China; 4 The First Affiliated Hospital of Jiangxi Medical College, Shangrao, Jiangxi, Province China

**Keywords:** Disease prevention, Eye manifestations

## Abstract

Chest pain patients without obstructive ischemic heart disease (IHD) have increased attention in the clinical practice as carrying higher cardiovascular (CV) risk and impaired life quality. Retinal vasculature is a novel but reliable risk factor of atherosclerosis and systemic vascular diseases. However, the association of retinal blood vessels and unobstructed IHD, as known as microvascular angina (MA) is poorly understood. This study compared retinal vascular structures of obstructive IHD and MA using spectral domain optical coherence tomography (SD-OCT) and full-width half-maximum (FWHM) methods to provide new risk predictive evidence of MA. Fundus vessels of 120 IHD patients, including 91epicardial IHD and 29 MA patients, and 66 control subjects were evaluated. Significant differences in the retinal arterial lumen diameter (RALD), retinal arterial outer diameter (RAOD), and arteriovenous ratio (AVR) have been found (P < 0.05). The severity of IHD was negatively correlated with diameters of RAOD, RALD and AVR (P < 0.05). In conclusion, there were significant differences in the retinal vascular structure between IHD patients and patients with MA. Thus, assessment of retinal vascular structure is suggested to evaluate CV risk of IHD patients, despite having no obstructive IHD.

## Introduction

With increases in the standard of living, the incidence of ischemic heart disease (IHD) has increased in recent decades, and it has become a disease that seriously endangers human health. IHD is a myocardial disease caused by myocardial ischaemia and hypoxia caused by changes in the coronary artery structure (e.g., stenosis, muscle bridge, dysplasia) or dysfunction (e.g., spasm), also known as ischaemic cardiomyopathy. microvascular angina (MA), also known as microvascular angina pectoris, is a relatively common cardiovascular disease. Due to the normal coronary angiography findings in MA, patients with recurrent angina symptoms have received increasing attention from clinicians in recent years. Studies have shown that MA has a good prognosis, but approximately 40% of patients are re-admitted for recurrent chest pain^1^, and these patients are more prone to myocardial infarction and stroke than the general population^2^. This finding suggests that MA not only seriously affects the quality of life of patients but also leads to repeated medical treatment, increasing the social burden for these patients. As a result, the need for early detection and diagnostic tools for MA is increasing. At present, retinal blood vessels are receiving more and more attention, which can be used as an important index to evaluate the risk of systemic vascular accidents. There is evidence that retinal vascular abnormalities are associated with clinical and subclinical manifestations of atherosclerosis and are risk factors for vascular disease^3^. The examination method is simple and convenient. Therefore, in clinical practice, it is necessary to assist in the diagnosis of IHD and to evaluate the development of the disease by observing changes in the morphology and structure of the retinal blood vessels.

As a type of rapidly developing fundus examination equipment in recent years, SD-OCT can be used for quantitative and qualitative analyses of retinal tissue structure. In this study, 120 patients with IHD were enrolled, and their retinal vessel-related data were obtained using SD-OCT and compared with coronary angiography results to explore the differences in retinal vascular structure between IHD patients and patients with MA.

## Materials and Methods

### Research population

From July 2016 to April 2018, 135 patients who were admitted to the Department of Cardiology at Quzhou People’s Hospital (Quzhou, Zhejiang Province) for suspected IHD were included in this study. Due to the unclear refractive medium and the patient’s request for termination of the examination, 15 patients withdrew from the study, and 120 patients were eventually enrolled. The selected patients underwent SD-OCT examination 1 day before coronary angiography. Additionally, they were routinely examined via electrocardiography and echocardiography, as well as fasting blood lipids (total cholesterol (TC), triglycerides (TG), high-density lipoprotein cholesterol (HDL-C), low-density lipoprotein cholesterol (LDL-C)) and other laboratory indicators. According to the results of routine electrocardiography, echocardiography and coronary angiography, the patients were divided into a IHD group and MA group. At the same time, 76 people without chest pain symptoms (matched according to age, gender, body mass index (BMI), smoking history, hypertension, diabetes and dyslipidaemia) were selected as the normal subjects. The routine electrocardiogram and cardiac ultrasonography were performed in these subjects. After the relevant contraindications were excluded, the exercise plate test was performed. Fasting blood lipids (TC, TG, HDL-C, LDL-C) and other laboratory indicators were assessed simultaneously with the eye SD-OCT examination. Among these subjects, 8 people withdrew due to unclear refractive medium, and 2 people withdrew due to a positive motion plate test. The final selection of 66 cases with negative motion plate test served as the control group.

Clinical data were obtained from all subjects, including age, gender, smoking history, history of hypertension, history of diabetes, and other clinical data were obtained from their hospital medical records. The study was approved by the research ethics committee of Quzhou People’s Hospital and was conducted in adherence to the tenets of the Declaration of Helsinki and relevant local guidelines and regulations. All the subjects involved in this study have been well explained and informed of the study purpose, study procedure and potential risk before taking part in the study. All the personal and clinical data obtained from patients are confidentially used for scientific research purpose only. Subjects are totally voluntary to take part in the study and preserve the rights to quit the study without any reasons, which would not affect the current and future clinical service of subjects. The study consent forms have been signed by subjects themselves. If the subject is under 18 years old, the consent form signed by parent or legal guardian is also required.

The fundus vessels could be clearly observed in all patients, except for those with low myopia (refractive error less than −3.00 dioptres), and there were no other eye-related diseases. No drugs that affected the diameter of blood vessels, such as angiotensin inhibitors and vasodilators, had been taken recently. People with severe liver and kidney dysfunction, thyroid disease, and cerebrovascular accidents or other neurological disorders, as well as recent trauma, surgery, or pregnancy, were excluded from the study.

### Definition of IHD

IHD patients have a known history of myocardial infarction and/or coronary angiography showing a primary branch anatomical stenosis ≥50% or a secondary branch stenosis ≥75%.

### Definition of MA

MA patients have typical symptoms of exhaustive chest pain and normal coronary angiography or coronary angiography showing primary branch stenosis <50% and/or secondary branch stenosis <75%. Chest pain patients induced by other cardiovascular diseases such as primary and secondary myocardial diseases, congenital heart disease, valvular heart disease are excluded from this cohort based on available echocardiogram and clinical history record.

### Coronary angiography

Coronary angiography was performed using a Philips 5000 digital subtraction angiography (DSA) machine (Royal Philips Inc., Netherlands), and the positions of the subjects were selected according to routine procedures using the Judkins method; other positions were added if necessary. Coronary artery lesions were measured using quantitative metrology-coronary angiography (QCA).

### Retinal blood vessel scanning and image acquisition

**Figure 1:**
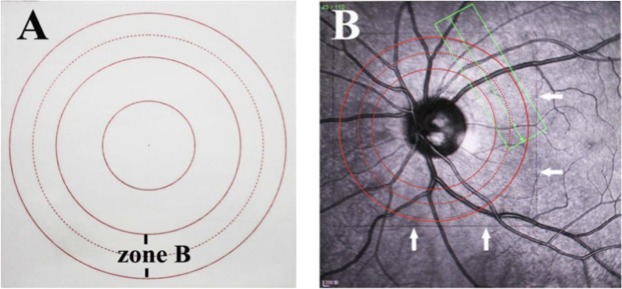
A concentric circle is drawn on transparent plastic film to determine the B area. According to the magnification of the fundus image on SD-OCT, the centre circle determines the diameter of the optic disc. Then, two concentric circles are drawn with one disc diameter and 1.5 disc diameters. The second circle and the third circle are in the B area. The blood vessels in zone B are scanned. The plastic film is attached with the positioning ring to the computer screen. The position is attached to the centre of the optic disc. The position of the B area is determined, and the blood vessels in the area are scanned.

**Figure 2:**
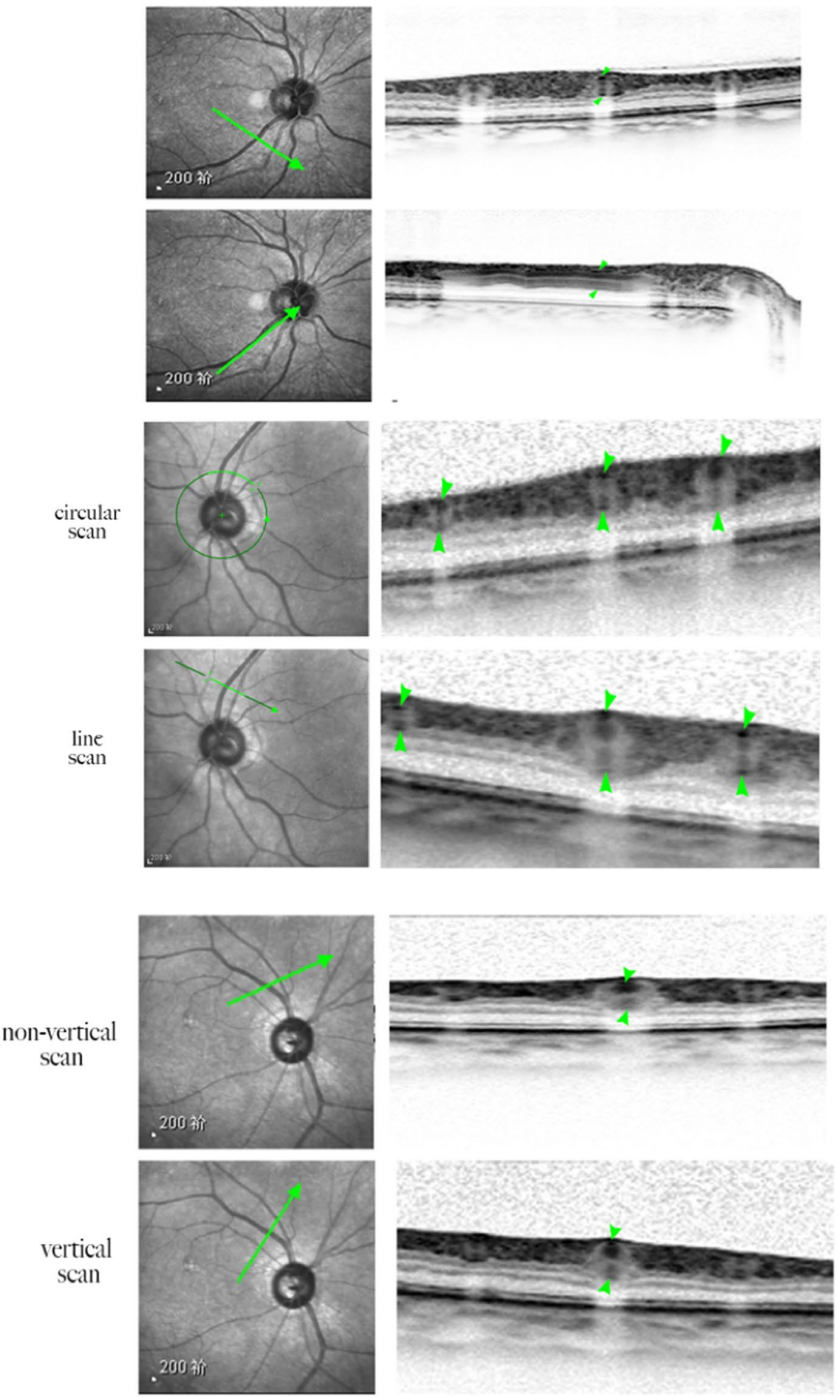
When the scan line coincides with the supraorbital retinal artery and vein, the image shows that the corresponding retinal blood vessels start from the optic disc and are located between the nerve fibre layer and the inner striate layer, and the upper and lower vessel wall structures are visible. When the scan line is perpendicular to the blood vessel, the cross-sectional structure of the retinal blood vessel can be obtained, and the upper and lower wall structures of the blood vessel and the blood flow image of the lumen are displayed. When the scanning line is not perpendicular to the blood vessel, the retinal vascular structure is blurred, and the image of the blood vessel wall is unclear. When the scan line is perpendicular to the blood vessel, the wall structure of the blood vessel is clearer than before. When a circular scan is used to scan the retinal arteries and veins of each quadrant, part of the vascular structure is not clearly displayed, and the linear structure is clearer than the circular scan.

The study was performed using a Heidelberg SD-OCT instrument (Heidelberg Engineering Inc., Heidelberg, Germany). First, it was determined that the scanning site was the B zone. Morphologically, the characteristics of the blood vessels in zone B are more in line with the description of small arteries and venules. Second, after the retinal blood vessels are emitted from the centre of the optic disc, there is less arteriovenous crossing and pulsation of the retinal artery in the B area, far from the optic disc. Measurement of the retinal arteriovenous diameter was not affected. The scan was completed before the vessel branching in the B zone region. If the blood vessel had branched before the B zone, it was scanned before it branched (Fig. 1). After the scan was completed, the vertical: horizontal ratio of the obtained OCT image was adjusted to 1:1 μm and enlarged to 800%, and the OCT image screenshot in the BMP image format was saved. Only the image showing the blood vessel wall could be selected for the next study. At least 5 clear OCT images were scanned for each blood vessel for analysis. We found through previous studies^4^ that different forms of retinal blood vessels can be obtained using different scanning methods (Fig. 2).

We confirmed that the inner diameters of the retinal arteries and veins obtained using the above method were close to the diameters of the retinal arteries and veins obtained through colour photography of the fundus, which demonstrates the feasibility of SD-OCT to measure the inner and outer diameters of the retinal vessels and its ability to obtain more vascular structural parameters (e.g., blood vessel wall thickness)^4,5^.

### Measurement of retinal vascular structural parameters based on the full-width half-maximum

The full-width half-maximum (FWHM) is a type of segmentation method for image edge recognition. The method has high accuracy when applied to the recognition of retinal blood vessel edges in SD-OCT images, and it is less affected by peripheral tissue image signals.Therefore, we use FWHM to obtain retinal blood vessel-related data.

**Figure 3:**
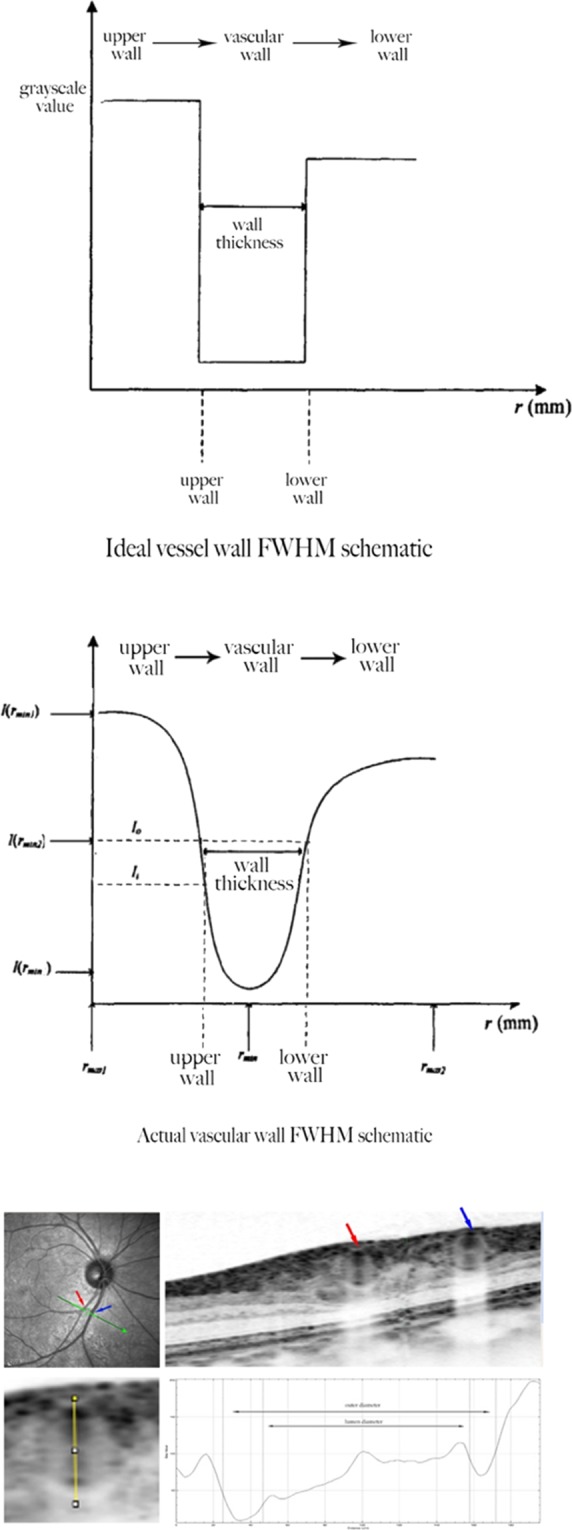
A line scan was performed across the vessel in zone B, and the line was aligned manually as perpendicular as possible to the running direction of the vessel. The line selection vertically crossed the middle of the upper and lower vessel walls to produce an intensity profile. The boundary points (arrow heads) were estimated at half-maximum intensity for each side of the two parabolas in the profile. The distance between the boundary points was calculated for the vessel outer and lumen diameters.

A clear retinal vascular structure image was obtained using the above method, and the longitudinal gradation curve of the target blood vessel was obtained using ImageJ software (National Institute of Health); the parabola of the two openings upward represent the upper and lower walls of the blood vessel (Fig. 3). After determining the edge of the vessel wall, the inner and outer diameters of the retinal vessels could be measured. Additionally, we obtained more retinal blood vessel-related parameters as follows: arterial wall thickness (AWT) = (RAOD-RALD)/2 and venous wall thickness (VWT) = [retinal venular outer diameter(RVOD)-retinal venular lumen diameter(RVLD)]/2. We measured the main temporal superior arteriole and venule in B zone of right eye as the temporal vessels were relatively larger without obvious branches,and located away from the optic papilla than the nasal side with less blood vessel pulsation effect during the measurement. Measurements of right eye and left eye have good correlation^6^. All operations were performed by experienced technicians. For all patients, each blood vessel was repeatedly measured three times by the same method and calculated as the average value. If measurement of right eye could not be performed (e.g., ungradable photographs from media opacities, or incomplete visualization of the zones surrounding the optic disc required for these measure- ments), the retinal vascular caliber of the left eye was measured instead.

Our previous studies have shown that the full-width half-maximum methods can significantly reduce the error of repeated measurements and improve the accuracy of vascular measurements compared with manual measurements^4,5^.

### Statistical methods

SPSS version 21.0 statistical software for Mac (SPSS, Chicago, IL, USA) was used for the analysis. Normal distribution of continuous datasets was assessed by Shapiro-Wilk test. One-way ANOVA and Chi-square test or Fisher’s exact test were performed to compare different between three groups. Correlation of disease severity and vessel measures was assessed by Spearman rank correlation test. α = 0.05 was used as the test level, and the difference was considered statistically significant at P < 0.05.

## Results

Table 1 shows the comparison of the three basic parameters and indicates that there were no significant differences in age, gender, diabetes, smoking, hypertension, dyslipidaemia and BMI among the three groups (P > 0.05).

**Table 1:** Clinical characteristics of the subjects.

Variables	Ischemic heart disease (n = 91)	Microvascular angina (n = 29)	Controls (n = 66)	P
Age (yr)	64.73 ± 10.15	64.55 ± 10.02	62.77 ± 8.21	0.422
Male sex,	64 (70.3%)	19 (65.5%)	37 (56.1%)	0.181
Diabetes,	39 (42.9%)	15 (51.7%)	30 (45.5%)	0.704
Current or ex-smoker	44 (48.4%)	14 (48.3%)	30 (45.5%)	0.932
Hypertension	62 (68.1%)	19 (65.5%)	36 (54.5%)	0.209
Dyslipidemia	27 (29.7%)	10 (34.5%)	17 (25.8%)	0.677
BMI (kg/m²),	23.98 ± 3.17	25.06 ± 2.82	23.63 ± 3.91	0.170

Data was displayed as mean ± standard deviation or numbers with percentages.

Body Mass Index(BMI).

Table 2 shows the results of the three groups of indicators. The findings show that there were no significant differences among the three groups in terms of RVLD or RVOD, (P > 0.05). There were significant differences among the three groups in terms of RALD, RAOD, and AVR (P < 0.05). After performing a pairwise comparison (LSD method), there was a significant difference between the IHD group and the control group in terms of RALD, RAOD, and AVR (P < 0.05).

**Table 2:** Difference of retinal vessel parameters in IHD, MA and normal subjects.

	Ischemic heart disease (n = 91)	Microvascular angina (n = 29)	Controls (n = 66)	F	P
RALD* (μm)	112.482 ± 14.686	114.503 ± 15.470	118.845 ± 12.498	3.934	0.021
RAOD* (μm)	145.278 ± 17.029	148.907 ± 18.682	153.055 ± 13.801	4.393	0.014
RVLD (μm)	153.321 ± 19.312	156.786 ± 18.270	153.071 ± 19.981	0.418	0.659
RVOD	184.368 ± 23.590	186.334 ± 20.974	182.414 ± 21.803	0.330	0.719
AVR*	0.798 ± 0.122	0.803 ± 0.093	0.850 ± 0.122	3.894	0.022
AWT (μm)	16.398 ± 2.936	17.202 ± 3.376	17.105 ± 2.411	1.579	0.209
VWT (μm)	15.524 ± 3.686	14.774 ± 3.489	14.671 ± 3.335	1.262	0.286

Note: * indicates the statistically significant difference. Data was displayed as mean ± standard deviation.

(P < 0.05). RAOD = retinal arterial outer diameter; RALD = retinal arterial lumen diameter; RVOD = retinal venous outer diameter; RVLD = retinal venous lumen diameter; AWT= arterial wall thickness; VWT = venous wall thickness.

Table 3 shows that the severity of IHD was negatively correlated with RALD, RAOD and AVR (P < 0.05). There was no correlation among RVLD, RVOD, AWT, and VWT (P > 0.05).

**Table 3:** Spearman’s analysis of coronary heart disease severity and retinal vessel parameters.

	Ischemic heart disease (n = 91	Microvascular angina (n = 29)	Controls (n = 66)	r	P
RALD (μm)	112.482 ± 14.686*	114.503 ± 15.470	118.845 ± 12.498	−0.172	0.019
RAOD (μm)	145.278 ± 17.029*	148.907 ± 18.682	153.055 ± 13.801	−0.198	0.007
RVLD (μm)	153.321 ± 19.312	156.786 ± 18.270	153.071 ± 19.981	−0.014	0.844
RVOD (μm)	184.368 ± 23.590	186.334 ± 20.974	182.414 ± 21.803	0.034	0.641
AVR	0.798 ± 0.122*	0.803 ± 0.093	0.850 ± 0.122	−0.156	0.034
AWT (μm)	16.398 ± 2.936	17.202 ± 3.376	17.105 ± 2.411	−0.136	0.065
VWT (μm)	15.524 ± 3.686	14.774 ± 3.489	14.671 ± 3.335	0.103	0.162

Data was displayed as mean ± standard deviation.

RAOD = retinal arterial outer diameter; RALD = retinal arterial lumen diameter; RVOD = retinal venous outer diameter; RVLD = retinal venous lumen diameter; AWT = arterial wall thickness; VWT = venous cross-sectional area.

## Discussion

With an ageing population and improvements in living conditions, the prevalence of cardiovascular and cerebrovascular diseases (IHD, hypertension, stroke) is increasing each year, becoming a primary disease and the most burdensome chronic disease that threatens human health and life^7^. Traditional cardiovascular and cerebrovascular disease risk factors (e.g., age, gender, blood pressure, diabetes, smoking) do not have the same predictive value in the elderly; thus, it is important to identify new risk factors. It is now increasingly recognized that small vessel disease (microvascular disease) may play a physiological role in the development of subclinical and clinical cardiovascular diseases^8^. For example, microvascular disease affecting the brain arterioles is associated with most cases of lacunar cerebral infarction defined by MRI^9^. Coronary microvascular dysfunction may explain the occurrence of myocardial ischaemia in patients without significant coronary atherosclerosis^10^. As the only blood vessels that can be directly observed in the body, retinal blood vessels serve as a window to provide a method for studying the early structural changes and pathological features of the human microcirculation. Therefore, this study aimed to explore the correlation between retinal blood vessels and cardiovascular diseases, such as IHD, and whether retinal vessels can be used as a reference for assessing the health status of people with suspected disease. Mohammad Hosein Dehghan observed fundus vascular changes in subjects using direct ophthalmoscopy, evaluated the degree of sclerosing of the fundus arteries according to the Scheie method, and performed coronary angiography. The Gensini score was used to evaluate the degree of coronary artery lesions^11^. In China, a study was conducted to compare the colour photographs of the fundus in 117 patients with IHD and 76 normal subjects. The study showed that the incidence of fundus arteriosclerosis in patients with IHD was higher than that in the normal population, resulting in coronary atherosclerosis of the heart. Hardening is associated with fundus arteriosclerosis^12^. However, the above studies are limited to qualitative studies of retinal vascular morphology, and the results vary between individuals. To perform more intuitive studies and evaluate retinal blood vessels, researchers began performing quantitative measurements of the retina. Parr and Hubbard *et al*. invented a method for the quantitative measurement of retinal blood vessels. In their study, semiautomatic vascular measurement software was used to open the fundus-centred fundus photographic image, and blood vessels in the annular region (0.5 D-1.0 DD from the edge of the optic disc) were measured. The 6 largest arterioles and venules were measured, and the central retinal artery was then converted. Corresponding data (e.g., the equivalent diameter of the retinal artery and equivalent diameter of the central retinal vein) were obtained^13^. The method is simple in operation and high in automation and efficiency. It has been adopted by many large epidemiological investigation agencies and has high reproducibility in the measurement process.

Frequency domain coherence tomography (SD-OCT) imaging is an important imaging examination system for fundus retinal tomography. It has the advantages of high precision, clear imaging, dynamic continuous tomographic analysis and being noninvasive^14^, and it can also track eye movements and lock the blood vessels to be measured when the eyeball rotates. We used SD-OCT to dissect the retinal blood vessels to obtain a clearer vascular tomographic image, and it is convenient to repeatedly locate the blood vessels. In the past, most researchers used the SD-OCT built-in ruler software to manually measure blood vessels. This method requires the measurer to judge the locations of the inner and outer boundaries of the vessel wall. The repeatability is poor, and the error is too large. Image segmentation technology is an important technology in medical image processing. It divides the image into several meaningful parts according to the characteristics of image texture, greyscale and colour. The nature of these parts may be similar or opposite, and the real purpose is to extract the “interesting” part of the image to provide relevant assistance for clinical diagnosis. The full-width half-maximum (FWHM) method is an edge-based segmentation method. The general idea underlying this method is to segment the recognition image by changing the grey value of the pixel at the edge of the area. This procedure makes the determination of blood vessel boundaries faster and more stable, and it is less sensitive to noise and adjacent tissue interference. Based on the above advantages, we used the FWHM to quantitatively analyse the retinal vascular structure.

The development of SD-OCT and image analysis techniques allows us to objectively measure retinal vessel diameters. Quantitative measurement of retinal vessel diameters greatly increases the understanding of the clinical significance and impact of systemic, environmental, and genetic factors on the retinal vasculature. The underlying mechanisms of retinal vessel diameter changes are still unknown, and changes in retinal vessel diameter may be the result of a combination of factors, including race, age, gender, inflammation, and other vascular factors. However, the standard reference levels for retinal vessel diameters, such as age, gender, and blood pressure levels, have not been determined. Standard reference levels play a crucial role in implementing these measurements in a clinical setting. There are many systematic and environmental effects on changes in the measurement of retinal arterioles and venules; therefore, it may be difficult to determine uniform criteria for different individuals or populations. In the adult population, it is difficult to explain the confounding effects of systemic diseases (e.g., hypertension and diabetes) on retinal vascular measurements^15^. Therefore, the purpose of this study was to investigate the correlation between retinal vessel diameter and the severity of coronary lesions, rather than to define the standard normal values of the retinal vessel diameter in each population.

The data indicate that approximately 20% to 30% of normal patients (assessed via coronary angiography) have typical angina symptoms^16^. In this study, the proportion of normal patients experiencing typical angina symptoms was 24%. Likoff first reported a case of typical angina-like pain with normal coronary angiography findings, which he later named “cardiac syndrome X”; it was suggested that these symptoms were mainly caused by coronary microvascular dysfunction^17^. Because these microvessels generally measure less than 500 μm in diameter, coronary angiography cannot identify their lesions. Therefore, the condition can also be called microvascular angina^16^. In fact, histopathological examination of endomyocardial biopsy revealed abnormalities in the small coronary structures in this population. These abnormalities include medial hypertrophy and luminal stenosis^18^. Retinal blood vessels are the only blood vessels in the whole body that can be directly observed. Retinal blood vessels are an effective indicator for evaluating systemic microvascular function. Because retinal blood vessels have similar anatomical and physiological characteristics as cerebral vessels and coronary circulation, their morphological changes can have a certain suggestive effect on cardiovascular diseases. Therefore, we can predict the status of systemic microvessels by measuring and evaluating parameters of retinal blood vessels. In this study, we measured retinal vascular findings in three groups. The MA group had lower RALD and RAOD values than the normal group but had higher RALD and RAOD values than the IHD group. Analysis of the arterial cross-sectional area also revealed that although there was no lesion in the aorta in this population, the retinal artery may have developed a pathological morphology similar to the coronary arteriole. Through research, we have found that this change is primarily reflected in the reduction in the diameter and cross-sectional area of the retinal artery.

Many studies have suggested that this change may be associated with impaired endothelial function. Endothelial cells have endocrine and metabolic effects. Active substances, such as endothelin (ET) and nitric oxide (NO), secreted by endothelial cells play a major role in vasoconstriction and relaxation. ET contracts blood vessels and promotes proliferation of vascular smooth muscle cells. NO has the effect of relaxing blood vessels and inhibiting the proliferation of vascular smooth muscle cells^19^. Therefore, dynamic changes in ET and NO have the effect of altering blood vessel morphology and regulating vascular tone. In 1995, for the first time, researchers suggested that elevated plasma levels of ET may be involved in the development of such diseases^20^. Some scholars in China have studied patients with typical angina symptoms and normal coronary angiography. When this group of patients is at rest, their plasma ET levels are not significantly different from those of normal patients. When patients exercised frequently, the ratio of plasma ET to NO increased at rest^21^. This result also suggests that vascular endothelial dysfunction may be involved in the pathogenesis of the MA. Based on previous research reports and the physiology of vascular disease, we speculate that this result is associated with endothelial dysfunction because there is no adrenal-related vasoconstriction in the retinal vessels to control the change of vascular tone^22^. The imbalance in the ratio of ET to NO leads to the proliferation of vascular smooth muscle cells as well as the hypertrophy of vessels and inhibition of diastolic function, which leads to smaller inner diameters, outer diameters and cross-sectional areas of the retinal artery.

Traditionally, it has been thought that changes in blood vessels are associated with only metabolically induced vascular degeneration. In 1999, Dr. Ross published a review in the *New England Journal of Medicine*, entitled “Atherosclerosis - an inflammatory disease”, which described how inflammation plays a key role in vascular changes^23^. This concept has been further developed over the past 20 years. In one experiment, the researchers injected mice with peroxide to find leukocyte exudation and increased retinal vein diameters in the retinal vessels^24^. It is currently believed that the widening of the retinal vein may be related to systemic inflammatory factors, ischaemia and hypoxia. Studies have found that larger retinal veins are associated with hsCRP, IL-6 and plasma fibre, suggesting that inflammation is a key cause of cardiovascular disease^25^. At the same time, dilated veins contribute to increased NO production and the release of inflammatory factors from vascular endothelial cells^26^, further leading to microcirculatory dysfunction. The Rotterdam study indicated that larger retinal vein diameters were independently associated with elevated levels of aortic atherosclerosis^27^. The Hoorn study reported that the relationship between the wide retinal vein diameter and the degree of arteriosclerosis became nonsignificant after controlling for cardiovascular risk factors^28^. In a recent study showed that the retinal microvascular structure in women with microvascular angina may be different from that in women with coronary artery disease(CAD)^29^. Considering the results of previous studies, our current study supports the hypothesis that retinal arteries are not associated with coronary atherosclerosis. Our analysis indicates that the associated reasons may be related to the different indicators observed. The above studies report equivalent retinal vein diameters. We measured the inner and outer diameters of each retinal vein in the B area. Additionally, the size of the sample and the difference in the study population impacted the study results.

The AVR is the ratio of the diameter of the retinal artery to the diameter of the accompanying venous blood vessel. It is a comprehensive index for evaluating retinal blood vessels. The Community Atherosclerosis Risk (ARIC) study suggests that this ratio provides information for predicting cardiovascular disease. The study also found that a small proportion of arteries and veins was associated with the incidence of IHD and stroke in a study of patients aged 51–72 years^30^, similar to the BMES (Australian Blue Mountains ophthalmology study). It has also been confirmed that a decrease in the ratio of retinal arteries and veins may lead to a higher risk of IHD. However, this indicator has corresponding limitations. It is not clear whether the arterioles or venules alone or together affect the AVR value. Therefore, some scholars have suggested that AVR should be reassessed by analysing retinal arterioles and venules separately^27^. In this study, we found that RALD and RAOD values in patients with IHD are smaller than those for the other two groups. After a comprehensive analysis of the corresponding RVLD and RVOD in each group, we still conclude that patients with IHD have lower AVR levels. This finding is in accordance with the above conclusions. Additionally, we observed symptomatic patients receiving coronary angiography and found that the normal group had a greater AVR value than the MA group, which suggests that microvascular disease may play a key role in disease development.

The innovation of this study is that we can obtain additional fundus morphological data, such as the thickness of the vessel wall. By comparing the thickness of the fundus arterial wall between 132 normal subjects and 106 hypertensive patients, previous Japanese researchers found that the mean arterial and venous wall thicknesses in hypertensive patients were significantly greater than those of age-matched subjects without hypertension. The thickness of the retinal arteriolar wall has also been suggested as an indicator that is sensitive to changes in the retinal arteriolar diameter in the diagnosis of hypertensive retinopathy^31^. Currently, there are no reports in any country on the relationship between IHD and retinal vessel wall. Therefore, in this study, we investigated the differences in retinal vessel wall thickness among three groups in the study population. Based on our findings, we have now concluded that there is no difference in retinal vessel wall thickness among these three groups. Subsequent research is required due to the small sample size.

Through the above studies, we found that there is a correlation between the degree of IHD and retinal arteries and veins. With the development and maturity of SD-OCT technology, as well as the convenience of retinal blood vessel observation, the association between retinal blood vessels and systemic vascular diseases can be better clarified. Relevance will also be revealed and developed. In future clinical work, we can obtain data on retinal vessel diameter and other data through SD-OCT to evaluate systemic microvascular conditions and provide strong support for the prevention and treatment of systemic vascular diseases.

This study has several limitations. First, because of the limitations of the conditions, patients had not undergone functional myocardial blood measurements such as index of microvascular resistance, coronary flow reserve and fractional flow reserve. Second, our sample size was small. The correlation between traditional cardiovascular and cerebrovascular disease risk factors and retinal vessel diameter must still be explored. The risk of diabetes, high blood pressure and other diseases and the length of the disease were not reflected in this study, and these factors will have an impact on the experimental results. Third, because the retinal vessels are close to the surface of the retina, it is difficult to distinguish the upper wall of the blood vessel from the tissue, resulting in a larger outer diameter of the blood vessel and a thicker vessel wall. Fourth, the FWHM method can reduce the measurement error. However, it is not fully automated; there is a possibility of error, and the steps are too cumbersome and inefficient. Currently, we are jointly developing more convenient and intelligent software that can perform a large number of blood vessel measurements, thereby improving the database. Fifth, retinal blood vessels demonstrate dynamic changes in morphology, which requires long-term follow-up. Analysis of the above shortcomings requires us to continue to increase the sample size in future research and perform long-term follow-up. We have also developed more intelligent image recognition technology to make the measurement of fundus vessels more precise, efficient and stable.

In conclusion, the IHD, MA and control groups showed significant differences in the RALD, RAOD, and AVR. SD-OCT and the FWHM method can accurately and noninvasively obtain fundus vascular data, which can be used to evaluate the degree of microvascular and coronary lesions.
