# Author Correction: A full-width half-maximum method to assess retinal vascular structural changes in patients with ischemic heart disease and microvascular angina

**DOI:** 10.1038/s41598-019-55182-y

**Published:** 2019-12-06

**Authors:** Bo Lun Xu, Wen Li Zhou, Tie Pei Zhu, Ke Yun Cheng, Yi Jie Li, Hai Jing Zhan, Li Gang Jiang, Yu Hua Tong

**Affiliations:** 1 Quzhou Central Hospital of Zhejiang Chinese Medical University, Quzhou People’s Hospital, Quzhou, Zhejiang Province China; 2 Department of Diagnostic Radiology, The University of Hong Kong, Hong Kong, SAR China; 3 Eye Center of Affiliated Second Hospital, Medical College of Zhejiang University, Hangzhou, Zhejiang Province China; 4 The First Affiliated Hospital of Jiangxi Medical College, Shangrao, Jiangxi, Province China

Correction to: *Scientific Reports* 10.1038/s41598-019-47194-5, published online 29 July 2019

In the original version of this Article, there was a repeated typographical error where the word “angina” was misspelled as “anginga”.

These errors have now been corrected in the HTML and PDF versions of the Article.

